# Enhancing children's environmental cognition, affect, and behavior through project-based STEM learning toward the SDGs

**DOI:** 10.3389/fpsyg.2026.1880850

**Published:** 2026-07-17

**Authors:** Ali Al-Barakat, Rommel AlAli, Amani Mohammed Al-Hosan, Muna Saad Alghamdi, Ali Abdullatif, Ashraf Zaher

**Affiliations:** 1 Department of Education, University of Sharjah, Sharjah, United Arab Emirates; 2 Faculty of Educational Sciences, Yarmouk University, Irbid, Jordan; 3 The National Research Center for Giftedness and Creativity, King Faisal University, Al-Ahsa, Saudi Arabia; 4 Department of Teaching and Learning, College of Education and Human Development, Princess Nourah bint Abdulrahman University, Riyadh, Saudi Arabia; 5 Department of Arabic Language, College of Arts, King Faisal University, Al-Ahsa, Saudi Arabia; 6 Translation, Authorship and Publication Center, King Faisal University, Al-Ahsa, Saudi Arabia

**Keywords:** early childhood, environmental education, project-based learning, psychology, stem education, sustainable development goals

## Abstract

**Objective:**

This study examined the impact of project-based environmental learning within the STEM framework on shaping children's environmental cognition, affect, and behavior in early childhood (aged 5–6 years), in alignment with the Sustainable Development Goals (SDGs), specifically SDG 6 (Clean Water and Sanitation), SDG 7 (Affordable and Clean Energy), SDG 12 (Responsible Consumption and Production), SDG 13 (Climate Action), SDG 14 (Life Below Water), and SDG 17 (Partnerships for the Goals).

**Methods:**

A mixed-methods design was employed, combining a quasi-experimental approach with a qualitative component. A convenience sample of 250 children from 12 early childhood education institutions in Saudi Arabia was randomly assigned to an experimental group (*n* = 125) and a control group (*n* = 125). The experimental group participated in structured STEM-based environmental projects embedded in authentic real-life contexts aligned with the targeted SDGs, whereas the control group received traditional instruction. Data were collected using an Environmental Knowledge Test, environmental affective and behavioral scales, and semi-structured interviews. Quantitative data were analyzed using descriptive statistics, independent samples *t*-tests, and multilevel modeling to account for institutional clustering. Qualitative data were analyzed using thematic analysis.

**Results:**

The findings revealed statistically significant differences between the experimental and control groups in favor of the experimental group across all cognitive, affective, and behavioral domains (*p* < 0.05). Multilevel modeling confirmed that these effects remained significant after controlling for institutional clustering across the 12 participating institutions. The qualitative findings supported and enriched the quantitative results, indicating that children internalized environmental values and translated knowledge into daily sustainable practices aligned with SDG-related behaviors.

**Conclusion:**

Project-based environmental learning within the STEM framework significantly enhances children's environmental cognition, affect, and behavior. The intervention effectively facilitates the transformation of environmental knowledge into meaningful real-life sustainable actions, thereby contributing to Education for Sustainable Development and advancing key Sustainable Development Goals, particularly SDG 6, SDG 7, SDG 12, SDG 13, SDG 14, and SDG 17.

## 1 Introduction

Rapidly intensifying environmental and social challenges, including climate change, biodiversity loss, and natural resource depletion, have increased the urgent need for innovative educational approaches capable of preparing environmentally responsible generations who can effectively address these pressing issues (UNESCO, 2021; Urválková and Surynková, 2021). In response to these global challenges, education is increasingly reconceptualized as a transformative process that extends beyond knowledge transmission to encompass the development of cognition, values, and behaviors aligned with sustainability principles (Ekasari et al., 2026; Imaduddin et al., 2025). Within this evolving perspective, environmental education in early childhood has gained significant attention as a foundational stage for shaping children's environmental awareness, attitudes, and behaviors (Avelar et al., 2019; Demaidi and Al-Sahili, 2021).

Building on this perspective, environmental education plays a crucial role in fostering sustainability-oriented mindsets among young learners (Ekasari et al., 2026). Its importance lies in its dual capacity to enhance environmental awareness and cultivate a sense of responsibility toward nature and society. Education for Sustainable Development (ESD) further strengthens this role by enabling learners to make informed decisions and act responsibly in relation to environmental, social, and economic dimensions (UNESCO, 2020, 2021). Consequently, early childhood environmental education contributes not only to cognitive development but also to emotional engagement and behavioral formation, which are essential for developing critical thinking skills and sustainable habits (Chawla, 2020; Watt and Frydenberg, 2025).

In this context, the integration of environmental education with Project-Based Learning (PBL) within STEM (Science, Technology, Engineering, and Mathematics) offers a more experiential and meaningful learning approach (Al-Barakat et al., 2025; Alotaibi et al., 2025). This integration bridges theoretical knowledge with real-world environmental challenges, enabling children to actively engage in inquiry-based and problem-solving activities (Alkair et al., 2023; Tarlochan et al., 2025). Through such learning experiences, children develop essential competencies such as collaboration, creativity, decision-making, and problem-solving. More importantly, this approach strengthens environmental sensitivity, empathy, and social responsibility, thereby linking knowledge with action and supporting the development of lifelong sustainable behaviors (Bell, 2010; Bybee, 2013; Tilbury, 2011).

Despite the growing recognition of Environmental Education (EE), STEM education, and Project-Based Learning (PBL), empirical research focusing on early childhood, particularly children aged 5–6 years, remains limited. Existing studies have predominantly targeted older students, teachers, or higher education contexts, leaving kindergarten settings underexplored. Moreover, previous research has often examined environmental knowledge, attitudes, or behaviors separately rather than investigating them as integrated dimensions of learning. This fragmentation limits a comprehensive understanding of how environmental learning interventions influence children's cognitive, affective, and behavioral development in a unified manner.

This limitation is further amplified in the Arab and Saudi context, where empirical research on integrated environmental STEM-PBL approaches in early childhood education is still emerging. In addition, a large proportion of existing evidence is derived from Western or Asian contexts, which may not adequately reflect the cultural, social, and educational characteristics that shape children's environmental perceptions and behaviors in Saudi Arabia. This contextual gap underscores the need for locally grounded empirical studies that respond to national educational priorities and sustainability agendas.

To address these gaps, the present study introduces three interrelated contributions. First, it focuses specifically on kindergarten children aged 5–6 years, a critical developmental stage that remains underrepresented in environmental STEM education research. Second, it adopts an integrated pedagogical framework that combines STEM and Project-Based Learning while simultaneously assessing cognitive (knowledge), affective (attitudes), and behavioral outcomes within a single coherent model. Third, it explicitly links early childhood environmental learning to the Sustainable Development Goals (SDGs), positioning sustainability as a measurable educational outcome rather than a purely conceptual framework.

Building on these contributions, the originality of this study lies in its contextual, methodological, and conceptual dimensions. Contextually, it addresses a significant gap in Saudi kindergarten education by focusing on an underexplored age group within environmental STEM-integrated learning environments. Methodologically, it employs a mixed-methods approach, integrating a quasi-experimental quantitative design with pre- and post-tests alongside appropriate statistical analyses to examine changes in children's environmental knowledge, affect, and behaviors related to sustainability. This is further strengthened by qualitative data that capture children's learning experiences, engagement, and interactions during STEM-based project activities. The integration of both methods enhances the depth, validity, and interpretive robustness of the findings through methodological triangulation.

Conceptually, the study advances sustainability education by integrating STEM and Project-Based Learning within early childhood education and explicitly embedding the Sustainable Development Goals (SDGs) into measurable learning outcomes. In doing so, sustainability is operationalized not as an abstract concept but as observable cognitive, affective, and behavioral competencies, thereby providing an empirically grounded framework for understanding sustainability development in early childhood.

Building on these considerations, the present study investigates the effectiveness of project-based environmental learning within a STEM framework in developing environmental knowledge, environmental affect, and sustainability-related environmental behaviors among Saudi kindergarten children aged 5–6 years. Unlike previous studies that have focused on isolated outcomes, older age groups, or non-Arab contexts, this study provides an integrated and context-specific contribution to early childhood sustainability education.

In order to operationalize this investigation and empirically examine the effects of the intervention across its targeted dimensions, the following research questions were formulated:

1. How does project-based environmental learning within the STEM framework influence children's environmental knowledge in relation to the SDGs compared to conventional teaching methods?
2. How does project-based environmental learning within the STEM framework influence children's environmental affect toward sustainability compared to conventional teaching methods?
3. How does project-based environmental learning within the STEM framework influence children's environmental behaviors related to sustainability compared to conventional teaching methods?

## 2 Literature review

Project-based environmental learning within Science, Technology, Engineering, and Mathematics (STEM) has been increasingly recognized as a contemporary pedagogical direction aligned with global sustainability agendas (Martínez-Borreguero et al., 2026; Veyis and Cigerci, 2025). Despite this growing attention, a critical examination of the literature reveals that research in this area remains conceptually fragmented and empirically uneven. Specifically, studies are distributed across four relatively independent domains: STEM education, project-based learning (PBL), environmental education (EE), and early childhood education (ECE), with limited evidence of systematic integration among them. This fragmentation has resulted in the absence of a unified pedagogical framework that explicitly connects instructional approaches with sustainability outcomes and the Sustainable Development Goals (SDGs), particularly in early childhood contexts (AlAli et al., 2025; Ardoin and Bowers, 2020).

Within STEM education research, the dominant emphasis is placed on developing cognitive and analytical competencies such as systems thinking, logical reasoning, problem-solving, and interdisciplinary integration (Bybee, 2013; Leung, 2023). Although these studies provide strong empirical support for cognitive enhancement, they are primarily conducted in upper educational levels, including primary, secondary, and tertiary education. Consequently, early childhood applications remain underdeveloped and often limited to simplified exposure activities rather than structured, inquiry-based engagement with real-world environmental problems. More importantly, STEM research rarely extends its focus beyond cognitive outcomes to include affective or behavioral dimensions, thereby limiting its contribution to sustainability education where values and actions are central.

Building on this limitation, the literature on project-based learning (PBL) emphasizes experiential, learner-centered instruction that promotes inquiry, collaboration, and active knowledge construction (Ardoin and Bowers, 2020; Bell, 2010; Ekasari et al., 2026; Imaduddin et al., 2025; Martínez-Borreguero et al., 2026; Veyis and Cigerci, 2025). However, despite its pedagogical strengths, PBL is frequently applied as a general instructional strategy rather than as a sustainability-oriented learning model. Most studies evaluate success in terms of engagement, motivation, or academic achievement, without explicitly linking project outcomes to environmental responsibility or behavioral transformation. This reflects a conceptual gap in which PBL is detached from broader sustainability frameworks such as ESD and the SDGs, despite its strong potential to operationalize them in practice.

In contrast, environmental education (EE) research provides substantial evidence that experiential and nature-based learning can significantly enhance environmental awareness, attitudes, and pro-environmental behaviors (Ardoin et al., 2020; Chawla, 2020). Nevertheless, a critical limitation in this body of literature is its tendency to treat cognitive, affective, and behavioral dimensions as separate constructs rather than interdependent components of a developmental learning system. Furthermore, EE studies are predominantly conducted with school-aged children or adolescents, while early childhood education, particularly the 5–6-year age group, remains significantly underrepresented. This limits the developmental sensitivity of existing findings, especially given that early childhood is considered a foundational stage for shaping long-term environmental dispositions.

When these three domains are analytically compared, a clear pattern of partial contribution emerges. STEM education primarily contributes cognitive structuring and analytical reasoning, PBL contributes pedagogical methodology and experiential learning design, and EE contributes environmental content and value formation. However, these components are rarely combined into a single integrated instructional model that simultaneously targets cognition, emotion, and behavior. This lack of integration reflects not only disciplinary separation but also methodological inconsistency in how learning outcomes are conceptualized, operationalized, and measured across studies.

From a broader theoretical perspective, Education for Sustainable Development (ESD) and the Sustainable Development Goals (SDGs) provide a comprehensive framework that integrates knowledge, values, skills, and action-oriented competencies (Tilbury, 2011; UNESCO, 2023). Despite this strong global policy framework, a significant gap persists between theoretical discourse and empirical classroom implementation. In particular, there is limited evidence on how SDG-related competencies can be effectively embedded within structured STEM–PBL interventions in early childhood settings. This gap is especially critical given that early childhood education is increasingly recognized as a key entry point for long-term sustainability learning, yet remains underutilized in empirical research.

Empirical studies that attempt partial integration of STEM, PBL, and environmental education (e.g., Ghosn-Chelala and Akar, 2021; Zhang et al., 2025) generally report positive outcomes, including improved environmental awareness, increased engagement, and enhanced conceptual understanding of sustainability issues. However, these studies are largely conducted in non-Arab contexts and focus predominantly on older learners, which significantly limits their developmental relevance and cultural transferability. As a result, there is a clear contextual gap in the applicability of findings to Saudi early childhood education, where cultural values, educational structures, and policy priorities differ substantially.

In the Arab educational context, existing research indicates that early childhood education systems remain largely traditional, with limited implementation of inquiry-based, experiential, or sustainability-oriented pedagogies (AlAli et al., 2025; Mansour et al., 2020). Moreover, environmental learning is frequently delivered in a fragmented manner, emphasizing isolated knowledge acquisition rather than the integrated development of cognitive, affective, and behavioral dimensions. This reflects a broader methodological limitation in regional research, whereby sustainability outcomes are not systematically conceptualized as multidimensional constructs. Consequently, opportunities for fostering comprehensive environmental competencies in early childhood remain limited.

Extending this contextual limitation, the international literature demonstrates similar patterns of fragmentation in the design and implementation of project-based environmental learning within a STEM framework. STEM education, project-based learning (PBL), and environmental education continue to function as largely separate domains rather than as an integrated pedagogical system. In addition, sustainability outcomes are often examined in isolation, with cognitive, affective, and behavioral dimensions treated as discrete variables rather than interrelated components of a unified learning process. This fragmentation is further compounded by the predominance of studies conducted in Western and Asian contexts, alongside a notable scarcity of empirical research addressing Arab and Saudi early childhood settings.

Taken together, these limitations underscore the need for more integrated and context-sensitive empirical investigations examining how STEM–PBL-based environmental learning can operate as a coherent pedagogical approach in early childhood education. Accordingly, the present study proceeds to the methodology section, where the research design, instruments, and procedures employed to examine the impact of this integrated approach on children's cognitive, affective, and behavioral sustainability-related outcomes are presented.

## 3 Method

### 3.1 Study design

The present study adopted a mixed-methods research design that integrates quantitative and qualitative approaches to provide a comprehensive understanding of both measurable outcomes and the contextual processes underlying children's learning experiences. Within the quantitative strand, the study employed a quasi-experimental pre-test–post-test control group design. The participants were assigned into two groups: an experimental group that received project-based environmental learning within the STEM framework, and a control group that was taught using conventional instructional methods. Prior to the intervention, a pre-test was administered to both groups to assess baseline equivalence across the three dependent variables: environmental knowledge, environmental affect, and environmental behavior. To statistically examine group equivalence at the pre-intervention stage, independent samples *t*-tests were conducted at a significance level of (*p* = 0.05). The results are presented in Table 1.

**Table 1:** Pre-test *t*-test results for group equivalence.

Instrument	Group	*N*	Mean	SD	*t*	df	*p*
Environmental knowledge test (EKT)	Experimental	125	7.98	1.76	−0.61	248	0.54
	Control	125	8.12	1.89			
Child environmental affective domain scale (CEADS)	Experimental	125	42.56	8.34	−0.54	248	0.59
	Control	125	43.12	7.98			
Child Environmental behavior scale (CEBS)	Experimental	125	44.25	7.87	−0.48	248	0.63
	Control	125	44.73	8.01			

The maximum possible score for the environmental knowledge test (EKT) is 25. The maximum possible score for both the child environmental affective domain scale (CEADS) and the child environmental behavior scale (CEBS) is 90.

**Figure 1:**
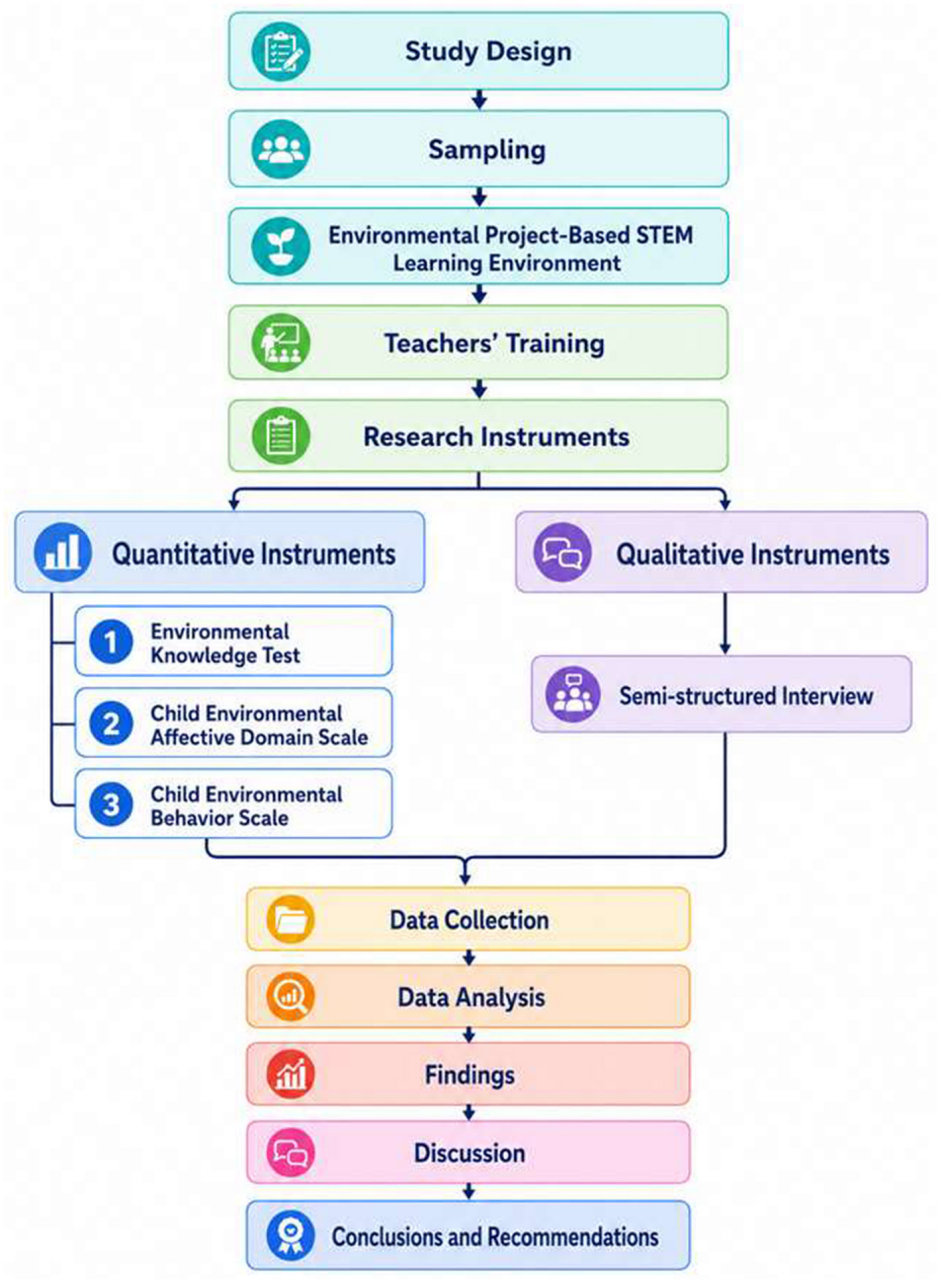
Research design.

Table 2 indicates that there are no statistically significant differences between the experimental and control groups at the pre-test stage across all measured variables (*p* > 0.05). This confirms baseline equivalence between the two groups in terms of environmental knowledge, affect, and behavior. Establishing this equivalence enhances the internal validity of the quasi-experimental design and ensures that any observed differences in the post-test phase can be attributed to the effects of the project-based environmental learning intervention within the STEM framework rather than pre-existing group differences. The overall structure of the research design is illustrated in Figure 1.

**Table 2:** Construct validity and reliability metrics for environmental knowledge, affective, and behavioral scales.

Instrument	Domain	Ω	CR	AVE	√AVE
Environmental knowledge test	Remembering	0.975	0.972	0.960	0.980
	Understanding	0.968	0.965	0.955	0.978
	Application	0.962	0.960	0.950	0.975
Child environmental affective domain scale (CEADS)	Environmental care and cleanliness	0.970	0.968	0.955	0.977
	Individual responsibility	0.975	0.973	0.960	0.980
	Participation and collaboration	0.965	0.962	0.950	0.975
	Pride and satisfaction	0.972	0.970	0.955	0.978
Child Environmental behavior scale (CEBS)	Water, energy, and waste management	0.975	0.972	0.960	0.980
	Participation in environmental activities	0.968	0.965	0.955	0.977
	Awareness of environmental activity importance	0.965	0.963	0.950	0.975
	Initiative for environmental actions	0.972	0.970	0.955	0.978

### 3.2 Study sample

A convenience sampling technique was initially employed to select 250 children aged 5–6 years from 12 early childhood education institutions in the Eastern Province of Saudi Arabia. This approach was adopted due to practical considerations, including institutional accessibility, administrative approval, and the willingness of schools and teachers to participate in implementing the project-based environmental learning intervention within the STEM framework. The selected institutions also provided suitable learning environments and resources necessary for conducting the study procedures.

Although the selection of institutions was based on convenience sampling, strict inclusion criteria were applied to ensure sample homogeneity. All participating children were enrolled in formal early childhood education settings within the same geographical region, which helped reduce contextual variability related to cultural, social, and environmental factors. In addition, restricting the sample to children aged 5–6 years ensured developmental consistency in cognitive, linguistic, and socio-emotional characteristics.

It is important to note that each participating institution included multiple classrooms (more than six sections in some cases). Therefore, a classroom-level random selection procedure was first applied within each institution, whereby one classroom was randomly selected to represent that institution in the study. This step ensured that the participating units were not arbitrarily chosen and reduced potential selection bias at the institutional level.

Following classroom selection, a second stage of randomization was conducted to assign participants into two equivalent groups. Specifically, children were randomly assigned to either the experimental group (*n* = 125), which received project-based environmental learning within the STEM framework, or the control group (*n* = 125), which continued with conventional instructional methods. This two-stage randomization procedure ensured equal probability of assignment and minimized selection bias at the participant level.

Finally, group equivalence was examined in terms of age distribution, educational level, and baseline performance levels (high, average, and low achievement categories). This combination of convenience sampling at the institutional level, random selection of classrooms, and random assignment of participants strengthened the internal validity of the study and enhanced the comparability between the two groups.

### 3.3 Environmental project-based stem learning environment

The environmental project-based learning environment within the STEM framework was designed as an integrative educational model aimed at enhancing early childhood education through the systematic development of cognitive, affective, and behavioral domains in alignment with the Sustainable Development Goals (SDGs). This design is based on the premise that effective environmental education extends beyond knowledge acquisition to include the development of values, emotions, and sustainable real-life practices, thereby fostering environmentally responsible and sustainability-oriented learners.

Building on this foundation, the learning environment integrated STEM disciplines within authentic environmental contexts. Science supported children's understanding of key environmental phenomena such as the water cycle, energy systems, natural resources, and ecological balance. Technology was incorporated through digital tools and interactive media to enhance visualization and engagement. Engineering was reflected in children's involvement in designing simple solutions to environmental problems, such as recycling systems and resource conservation strategies. Mathematics was embedded through activities involving measurement, comparison, classification, and interpretation of environmental data. Collectively, these interdisciplinary components were linked to real-life sustainability challenges such as pollution reduction, responsible consumption, and climate action, ensuring direct alignment with the SDGs.

In continuation of this integrative design, the learning environment was structured around three interrelated dimensions: cognitive, affective, and behavioral. The cognitive dimension focused on developing children's understanding of environmental concepts and cause-effect relationships. The affective dimension aimed to strengthen emotional connection with nature and foster positive environmental attitudes. The behavioral dimension emphasized translating knowledge and emotions into sustainable practices within school and home contexts, ensuring real-life application and continuity of learning.

Guided by these dimensions, the project-based learning process was organized around an overarching objective: enabling children to perceive the environment as an interconnected system, develop environmental awareness, and adopt sustainable behaviors. This objective guided the selection of content, learning activities, and assessment procedures, ensuring full alignment with the SDGs. In particular, the program was structured around key goals, including SDG 6 (Clean Water and Sanitation), SDG 7 (Affordable and Clean Energy), SDG 12 (Responsible Consumption and Production), SDG 13 (Climate Action), SDG 14 (Life Below Water), and and SDG 17 (Partnerships for the Goals).

Within this framework, a core instructional unit titled “*The Environment Around Us”* was developed as the foundation of learning content. This unit introduced essential environmental concepts such as water, energy, waste, natural resources, and pollution in a structured progression from simple to more complex ideas, ensuring developmental appropriateness for early childhood learners.

To operationalize learning, a series of environmental projects was designed based on real-life problems familiar to children. These projects followed the phases of project-based learning: observation, questioning, planning, implementation, and reflection. Examples included monitoring classroom water use, designing simple recycling solutions, and participating in school-based environmental awareness activities. These experiences actively engaged children in inquiry, collaboration, and problem-solving, thereby strengthening their sense of responsibility toward environmental protection.

To further support learning, multiple instructional strategies were systematically integrated. Storytelling and character-based learning presented environmental concepts through child-friendly narratives featuring characters such as “Water Drop,” “The Sun,” and “The Plant,” making abstract concepts more meaningful and emotionally engaging. Inquiry-based learning fostered curiosity and exploration, while cooperative learning promoted communication and shared responsibility. Experiential learning enhanced understanding through hands-on activities such as recycling and water conservation. Problem-based learning introduced simple environmental challenges requiring children to propose solutions, thereby strengthening critical thinking and creativity. Additionally, gamification and digital tools increased engagement, while reflective learning through guided discussions supported children in expressing their understanding, emotions, and behavioral intentions.

Supporting these strategies, diverse instructional media such as visual illustrations, short videos, audio elements, and interactive digital content were employed to enhance conceptual understanding and accommodate diverse learning styles. These tools also increased engagement and supported active participation in learning activities.

Consistent with a child-centered approach, the learning environment positioned children as active constructors of knowledge rather than passive recipients. Through exploration, experimentation, collaboration, and decision-making, children gradually developed environmental understanding, emotional engagement, and responsible behavioral patterns. The integration of school- and home-based activities further ensured continuity and real-life application of sustainable behaviors.

Finally, to ensure the quality and rigor of the program, the learning environment and instructional design were reviewed by a panel of experts in environmental education, early childhood education, and educational technology. Based on their feedback, necessary refinements were made to ensure developmental appropriateness, instructional coherence, and alignment with sustainability competencies.

### 3.4 Teachers training and pilot implementation

Following the validation phase, a structured teacher training program was conducted to prepare teachers in the experimental group for the effective implementation of environmental project-based learning within the STEM framework. The training comprised 24 instructional hours delivered over 3 weeks (8 h per week), and focused on developing teachers' competencies in facilitating environmental projects, managing learner-centered activities, and supporting children's engagement in inquiry, collaboration, and real-world problem-solving.

Building on this preparatory phase, a pilot implementation was subsequently conducted to test the practicality of the designed environmental projects within STEM-based instructional units. During this phase, sustainability-focused topics such as water conservation and responsible resource use were integrated into classroom activities. Children were actively engaged in structured inquiry processes, including observing environmental problems, collecting simple data, proposing solutions, and participating in collaborative environmental actions. This stage strengthened the connection between conceptual understanding and real-life environmental practices.

During the pilot implementation, several practical challenges emerged that required instructional adjustments to ensure effective delivery. One key challenge was the variation in teachers' prior familiarity with project-based learning approaches, which initially affected the consistency of implementation. This issue was addressed through intensive pre-implementation training and continuous in-class pedagogical support.

In addition, some children demonstrated difficulty in maintaining attention during open-ended project phases due to their limited prior exposure to inquiry-based learning environments. To address this, learning activities were reorganized into clear sequential stages, planning, implementation, and reflection, with structured transitions between phases. This adjustment improved children's engagement, focus, and motivation throughout the learning process.

Overall, these refinements contributed to a more structured, developmentally appropriate, and inclusive implementation process. As a result, all participants were able to benefit effectively from the environmental project-based learning environment within the STEM framework, which supported the holistic development of children's cognitive understanding, affective engagement, and behavioral competencies in alignment with sustainability education objectives.

### 3.5 Research instruments

#### 3.5.1 Environmental knowledge test

The Environmental Knowledge Test was developed as a comprehensive instrument to assess children's environmental knowledge and the extent to which it can be enhanced through different instructional approaches, particularly project-based learning within the STEM framework compared to conventional teaching methods.

The test consists of 25 multiple-choice items distributed across three cognitive levels to capture different dimensions of thinking. The first level, *Remembering*, includes ten items designed to assess children's ability to recall basic environmental facts and concepts related to water conservation, energy, waste management, and climate change. The second level, *Understanding*, consists of eight items that measure children's ability to interpret and explain environmental concepts and relate them to real-life situations, reflecting their comprehension of sustainable practices. The third level, *Application*, includes seven items that assess children's ability to apply environmental knowledge in practical contexts, such as identifying solutions to water leakage, practicing recycling, using renewable energy, and engaging in community-based environmental actions.

In alignment with the Sustainable Development Goals (SDGs), all items are linked to relevant goals, including SDG 6 (Clean Water and Sanitation), SDG 7 (Affordable and Clean Energy), SDG 12 (Responsible Consumption and Production), SDG 13 (Climate Action), and SDG 14 (Life Below Water). Each correct response is awarded one point, resulting in a maximum possible score of 25, which allows for statistical comparison between the experimental and control groups.

#### 3.5.2 Child environmental affective domain scale (CEADS)

The Child Environmental Affective Domain Scale (CEADS) was developed to assess children's emotional engagement with the environment and to examine the extent to which their feelings, attitudes, and environmental values can be influenced through different instructional approaches, particularly project-based environmental learning within the STEM framework compared to conventional teaching methods.

The scale consists of 30 statements distributed across four affective domains that collectively represent key dimensions of children's environmental affect. The first domain, *Environmental Care and Cleanliness*, includes eight statements measuring children's concern for environmental cleanliness and protection, including their attitudes toward littering, pollution, and maintaining clean school and community environments. This domain aligns with SDG 6 (Clean Water and Sanitation) and SDG 14 (Life Below Water), as environmental care contributes directly to pollution reduction and ecosystem protection.

Building on this dimension, the second domain, *Individual Responsibility*, comprises eight statements assessing children's sense of personal responsibility toward sustainable behaviors such as conserving water, saving energy, and protecting natural resources. This domain is linked to SDG 12 (Responsible Consumption and Production) and SDG 13 (Climate Action), reflecting the role of individual behavior in promoting environmental sustainability.

The third domain, *Participation and Collaboration*, includes seven statements evaluating children's willingness to participate in environmental activities, collaborate with peers, and contribute to environmental awareness initiatives within their learning environment. This domain is associated with SDG 11 (Sustainable Cities and Communities) and SDG 17 (Partnerships for the Goals), emphasizing the importance of collective engagement in sustainability efforts.

Complementing the previous domains, the fourth domain, *Pride and Satisfaction in Environmental Actions*, consists of seven statements measuring children's feelings of pride, enjoyment, and intrinsic motivation when engaging in environmentally responsible behaviors and observing their positive outcomes. This domain aligns with SDG 13 (Climate Action) and SDG 15 (Life on Land), highlighting the importance of positive emotional reinforcement in sustaining environmentally responsible behavior.

Given the young age of participants (5–6 years), the administration of the scale was carefully adapted to ensure developmental appropriateness and response validity. Although the scale was designed using a simplified three-point Likert format (Agree = 3, Sometimes = 2, Disagree = 1), it was not administered as a written self-report instrument. Instead, it was implemented through a guided oral interview format supported by visual aids such as pictorial representations and smiley-face scales to facilitate children's comprehension and engagement.

Each item was read aloud by the researcher using simple, age-appropriate language, and children responded by selecting the corresponding visual or verbal option. To ensure comprehension, practice examples were provided prior to the actual administration to familiarize children with the response procedure. In addition, teachers were present during administration to provide clarification when necessary, without influencing children's responses.

This multi-modal administration approach (oral explanation, visual support, and guided response) enhanced children's understanding of the items and strengthened the validity and reliability of their responses. Total scores ranged from 30 to 90, with higher scores indicating stronger positive environmental affect.

#### 3.5.3 Child environmental behavior scale (CEBS)

The Child Environmental Behavior Scale (CEBS) was developed to assess children's pro-environmental behaviors in daily life and to examine the extent to which these behaviors can be enhanced through different instructional approaches, particularly project-based environmental learning within the STEM framework compared to conventional teaching methods.

The scale consists of 30 items distributed across four behavioral domains representing key aspects of sustainable environmental behavior in early childhood. The first domain, *Water, Energy, and Waste Management*, includes eight items assessing children's environmentally responsible practices, such as turning off water taps, saving electricity, reducing unnecessary paper use, and sorting recyclable materials. This domain aligns with SDG 6 (Clean Water and Sanitation), SDG 7 (Affordable and Clean Energy), and SDG 12 (Responsible Consumption and Production), reflecting practical engagement in sustainable resource management.

Extending this behavioral dimension, the second domain, *Participation in Environmental Activities*, comprises eight items measuring children's involvement in environmental initiatives such as planting activities, classroom or school clean-up campaigns, and collaborative sustainability projects. This domain is linked to SDG 11 (Sustainable Cities and Communities) and SDG 17 (Partnerships for the Goals), emphasizing cooperation and collective environmental action.

The third domain, *Responsibility and Awareness*, includes seven items assessing children's awareness of the environmental consequences of their actions and their sense of responsibility toward protecting natural resources. This domain is associated with SDG 12 and SDG 13 (Climate Action), highlighting behavioral awareness in relation to sustainability and environmental protection.

Finally, the fourth domain, *Initiative and Pride in Environmental Actions*, consists of seven items measuring children's proactive environmental behaviors, independent initiatives, and feelings of pride when contributing to environmental protection. This domain aligns with SDG 13 and SDG 15 (Life on Land), reflecting intrinsic motivation and environmental stewardship.

Given the young age of participants (5–6 years), the administration of the CEBS was carefully adapted to ensure developmental appropriateness and measurement validity. Although the scale is structured as a simplified three-point Likert-type format (Always = 3, Sometimes = 2, Rarely = 1), it was not administered as a written self-report instrument. Instead, it was implemented through a guided oral interview format supported by visual aids and concrete behavioral examples to facilitate children's comprehension.

Each item was read aloud by the researcher using simple, age-appropriate language, and children responded by selecting the option that best represented their usual behavior using visual cues corresponding to each response category. Prior to the main administration, practice examples were provided to ensure that children understood both the meaning of the items and the response format.

To further enhance response accuracy and reduce misunderstanding, teachers were present during administration to provide clarification when necessary, without influencing children's responses. This multi-modal approach (oral explanation, visual support, and guided response) was designed to align with the cognitive and linguistic developmental level of early childhood learners and to improve the reliability of the collected data. Total scores ranged from 30 to 90, with higher scores indicating stronger and more consistent pro-environmental behaviors.

### 3.6 Semi-structured interview

The semi-structured interview in this study aimed to provide an in-depth understanding of the impact of project-based environmental learning within the STEM framework on children's affective and behavioral domains related to sustainability. Specifically, it sought to explore children's emotional engagement with environmental issues and the extent to which participation in project-based learning activities influenced the adoption of sustainable behaviors in their daily lives. This qualitative approach was chosen to complement the quantitative assessment of learning outcomes aligned with the Sustainable Development Goals (SDGs), offering a richer and more contextual understanding of the effects of the instructional intervention.

Interviews were conducted with all children in the experimental group to capture their perceptions, experiences, and reflections regarding participation in environmental projects. The interview guide was developed based on the researchers' expertise in early childhood education, environmental education, and STEM-based learning, ensuring its alignment with the study's objectives in examining both affective and behavioral domains.

The instrument was validated through a rigorous expert review, resulting in the adoption of eight main questions representing the core themes of the interview. These questions addressed the affective domain by exploring children's self-reported environmental care and cleanliness, individual responsibility, participation and collaboration, and pride and satisfaction. In addition, the behavioral domain was examined through self-reported environmental behaviors, including water, energy, and waste management, participation in environmental activities, responsibility and awareness, and initiative and pride in performing sustainable actions.

Follow-up (probing) questions were included to ensure comprehensive responses and to gain deeper insights into children's experiences and environmental behaviors. The instrument was also pilot-tested with a sample of children outside the study population to confirm its clarity, age-appropriateness, and overall reliability.

This semi-structured interview design allowed for flexibility in exploring individual responses and experiences, providing a rich understanding of how project-based environmental learning within the STEM framework influences children's affective and behavioral development. Furthermore, it facilitated the integration of qualitative and quantitative findings, thereby enhancing the comprehensiveness, validity, and interpretive strength of the study's results.

### 3.7 Validity and reliability

To ensure the rigor and accuracy of the study, validity and reliability procedures were conducted for the three research instruments: the Environmental Knowledge Test (EKT), the Child Environmental Affective Domain Scale (CEADS), and the Child Environmental Behavior Scale (CEBS). These procedures were designed in accordance with the developmental characteristics of children aged 5–6 years.

#### 3.7.1 Content validity

Content validity was established through review by a panel of seven experts in environmental education, early childhood education, STEM pedagogy, and educational assessment. The experts evaluated the alignment of the items with the study domains, the Sustainable Development Goals (SDGs), and the developmental level of the children. Minor revisions were subsequently made to improve clarity, linguistic appropriateness, and alignment with the study objectives.

#### 3.7.2 Face validity

Face validity was assessed through a pilot administration involving 25 children from the target age group. This phase examined the clarity of instructions, item comprehensibility, sequencing, and completion time. Based on observations during administration, minor modifications were introduced to simplify some items and improve readability and ease of response.

#### 3.7.3 Internal consistency reliability

**Figure 2:**
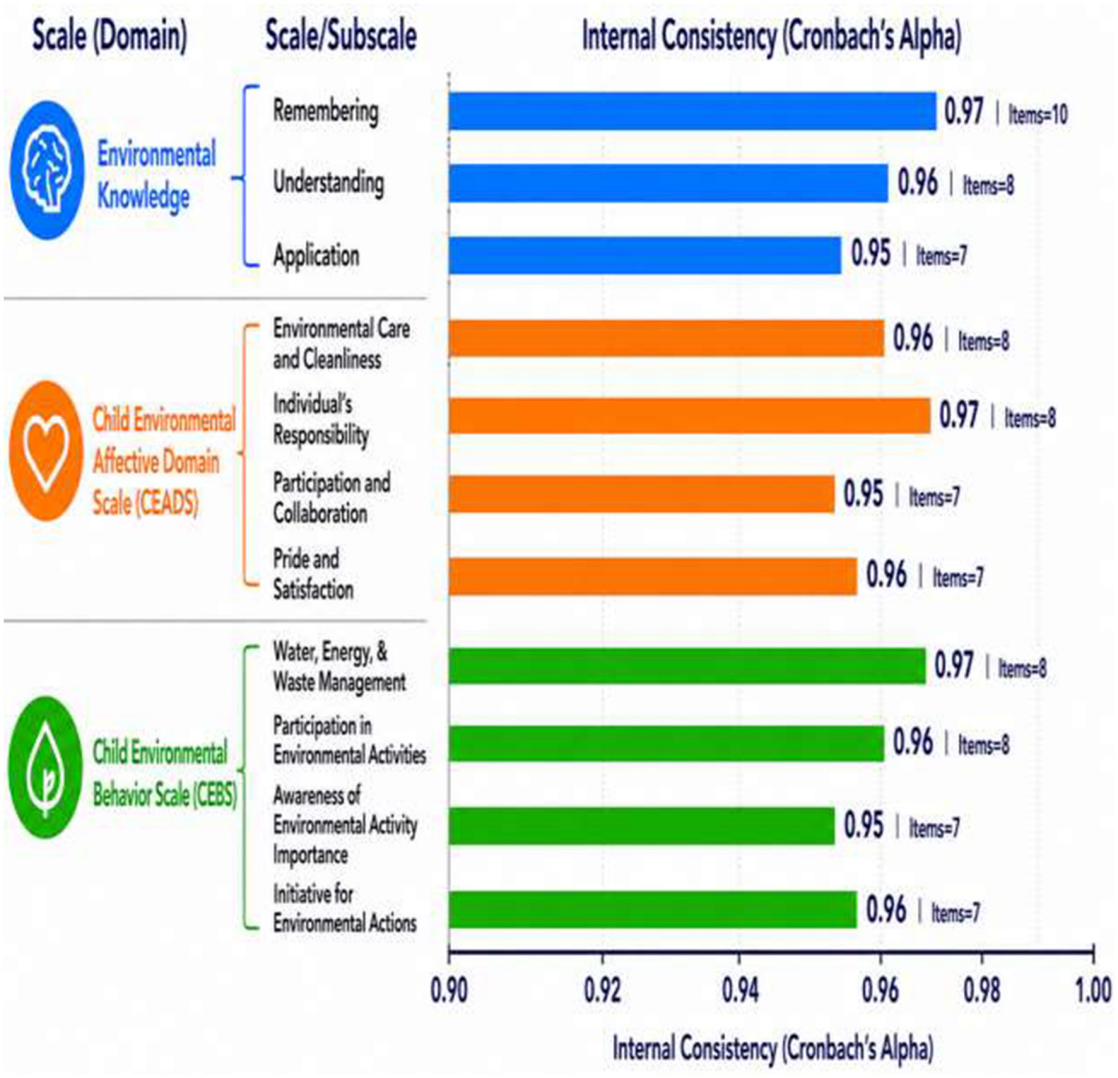
Internal consistency (Cronbach's Alpha) for environmental knowledge, affective, and behavioral scales.

Following the validity procedures, internal consistency reliability was examined using Cronbach's Alpha coefficients to assess the consistency of the items within each instrument and domain. The results indicated satisfactory reliability levels for all instruments, confirming their suitability for measuring children's environmental knowledge, affective engagement, and pro-environmental behaviors. Figure 2 presents the Cronbach's Alpha coefficients for all instruments and domains.

Figure 2 shows that all Cronbach's alpha values substantially exceed the recommended threshold of 0.70, indicating excellent internal consistency across all instruments and domains. These findings confirm that the items within each scale reliably measure their intended constructs. For instance, the exceptionally high reliability of the *Individual Responsibility* domain in the CEADS (α = 0.97) suggests that children's responses related to environmental responsibility are highly consistent and reflect a stable underlying construct. Likewise, the CEBS demonstrates strong internal consistency across its domains, supporting the scale's suitability for reliably and coherently assessing children's real-life pro-environmental behaviors.

#### 3.7.4 Construct validity and additional reliability measures

Construct validity and additional reliability metrics were evaluated using Omega (ω), Composite Reliability (CR), Average Variance Extracted (AVE), and the square root of AVE (√AVE). This was presented in Table 2.

Table 2 demonstrates strong construct validity across all instruments. The high Omega and Composite Reliability coefficients indicate that each domain measures a coherent and distinct latent construct, while the Average Variance Extracted (AVE) and √AVE values provide strong evidence of convergent validity. For example, the *Water, Energy, and Waste Management* domain within the CEBS showed a high level of internal consistency (Ω = 0.975), confirming that children's environmental practices represent a stable and reliably measurable construct.

Overall, the Environmental Knowledge Test (EKT), the Child Environmental Affective Domain Scale (CEADS), and the Child Environmental Behavior Scale (CEBS) demonstrated strong psychometric properties, providing reliable, valid, and interpretable data for quantitative analysis. These findings strengthened the accuracy of comparisons between the experimental and control groups and supported the study's objective of examining the effectiveness of project-based environmental learning within the STEM framework compared with conventional instructional methods.

In addition to the quantitative instruments, procedures were also implemented to establish the validity and reliability of the qualitative interview protocol. Initially, the interview schedule consisted of 13 open-ended questions. Following expert review, several revisions were introduced to improve clarity, precision of wording, and alignment with the study objectives. These modifications included simplifying the language to ensure developmental appropriateness for young children and restructuring the interview into eight main questions representing the core themes of the study.

To further strengthen the quality of the interview instrument, the revised schedule was evaluated by a panel of experts in early childhood education, science education, digital technology, and educational measurement and evaluation. This process ensured both face and content validity while confirming alignment with the study objectives. Based on the experts' feedback, additional refinements were made to the wording of follow-up prompts to ensure that each question effectively elicited responses related to children's environmental perceptions, emotional engagement, and sustainability-related behaviors.

Following the expert validation process, a pilot study was conducted with eight children who were not included in the main study sample. This phase resulted in minor refinements related to question wording, sequencing, and overall comprehensibility. These adjustments improved the clarity and consistency of children's responses and ensured that the interview questions were suitable for the target age group.

Finally, the reliability of the interview data was strengthened through the use of a semi-structured interview format, which ensured consistency across participants while allowing flexibility for deeper exploration through follow-up questions when necessary. Collectively, these procedures enhanced the trustworthiness and credibility of the qualitative data and ensured its alignment with the objectives of the study.

### 3.8 Data collection

Prior to data collection, teachers assigned to the experimental group participated in structured training sessions on the implementation of project-based environmental learning within the STEM framework. This preparatory phase was designed to ensure consistency in intervention delivery across participating classrooms and to support developmentally appropriate facilitation of learning activities for young children.

Following teacher preparation, baseline data were collected through the administration of pre-test and pre-assessment measures to both the experimental and control groups. To accommodate the developmental characteristics of children aged 5–6 years, all instruments were administered individually by the researchers, who read each item aloud to ensure comprehension and minimize the influence of reading ability on performance. The same administration procedures and testing conditions were maintained during the post-intervention assessment phase to ensure consistency and support the validity of pre–post comparisons.

The intervention was subsequently implemented over a 4-month period (January–April 2025), comprising five instructional sessions per week, each lasting approximately 45 min, for a total of 60 sessions. During this period, children in the experimental group engaged in a series of structured environmental projects designed to promote sustainability-related cognitive, affective, and behavioral competencies. The instructional activities integrated hands-on experiences with multimedia resources to enhance engagement, support active learning, and accommodate diverse learning preferences.

Upon completion of the intervention, post-test assessments were administered using the same instruments, procedures, and conditions employed during the pre-test phase. Maintaining identical assessment conditions was intended to ensure valid comparisons and provide an accurate measure of changes in children's environmental knowledge, affective engagement, and environmental behavior.

To complement the quantitative findings and gain deeper insight into children's learning experiences, qualitative data were collected through semi-structured interviews with participants in the experimental group. The interviews explored children's perceptions of their participation in environmental projects, their emotional engagement with environmental issues, and their application of sustainable practices in everyday contexts. In order to capture the full range of experiences generated by the intervention, all children in the experimental group were invited to participate in the interview process.

Accordingly, semi-structured interviews were conducted with all 125 children in the experimental group rather than with a selected subsample. This decision was informed by both methodological and educational considerations. From a methodological perspective, preliminary analysis indicated that data saturation was achieved after approximately 55 interviews, as responses became increasingly repetitive and no substantially new themes emerged, particularly within the affective and behavioral domains. Nevertheless, the interviews were continued with all participants to ensure comprehensive representation of children's experiences and to avoid excluding potentially meaningful individual perspectives.

From an educational perspective, children demonstrated a high level of motivation and willingness to participate in the interview process. Many expressed enthusiasm when discussing their environmental projects and experiences, and a positive sense of engagement emerged as children eagerly shared their ideas and accomplishments. This level of participation was facilitated by the supportive classroom climate and the strong rapport established among the researchers, teachers, and children. As a result, the interviews were experienced as enjoyable learning conversations rather than formal assessment activities.

To further support authentic participation, interviews were conducted in a friendly, interactive, and informal manner appropriate for children aged 5–6 years. The interview sessions were intentionally brief and embedded within the natural classroom routine to minimize fatigue and reduce potential anxiety. Age-appropriate language, visual prompts, and guided questioning techniques were employed to facilitate understanding and encourage spontaneous expression.

This child-centered approach enabled the collection of rich qualitative data while preserving the comfort and engagement of participants. Importantly, extending data collection beyond the point of saturation was not intended to increase the volume of data, but rather to enhance inclusiveness and ensure that every child had an opportunity to express his or her experiences within a supportive educational environment.

### 3.9 Data analysis

The quantitative data obtained from the three instruments, the Environmental Knowledge Test (EKT), the Child Environmental Affective Domain Scale (CEADS), and the Child Environmental Behavior Scale (CEBS), were analyzed using SPSS (version 30). Descriptive statistics, including means and standard deviations, were computed for both the experimental and control groups. To examine the effect of the intervention, independent samples *t*-tests were conducted at a significance level of (*p* = 0.05), allowing for the comparison of group differences in children's environmental knowledge, affective engagement, and behavioral outcomes.

**Figure 3:**
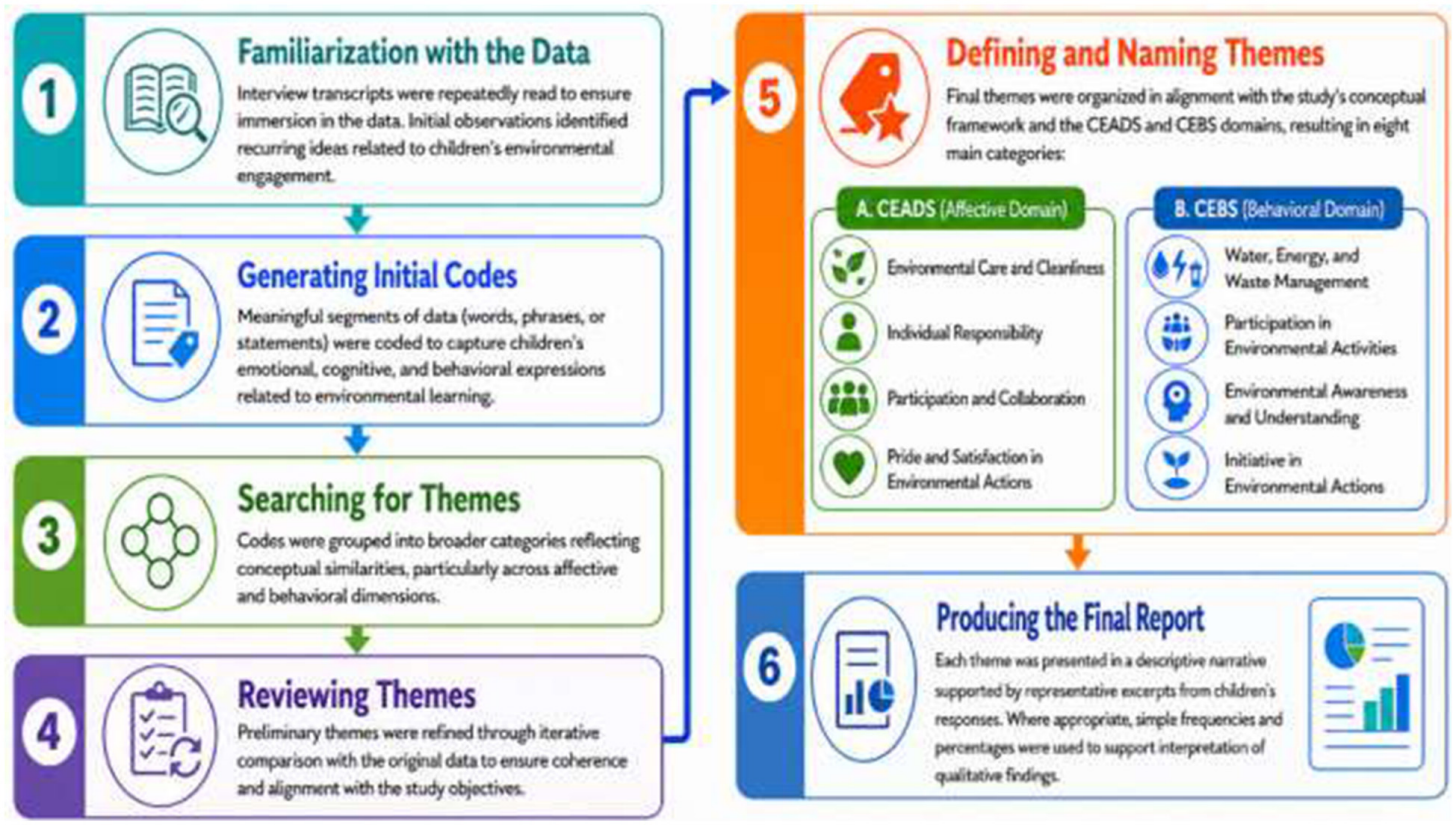
Thematic analysis process.

For the qualitative data collected through semi-structured interviews, thematic analysis was conducted following the approach of (Braun and Clarke 2006), supported by principles of grounded theory. This method enabled systematic identification of patterns emerging from children's responses and experiences. The analysis proceeded through six sequential stages as illustrated in Figure 3.

Figure 3 illustrates the thematic analysis process employed in this study. The analysis proceeded through six sequential stages, beginning with familiarization with the data and culminating in the production of the final analytical report. First, interview transcripts were repeatedly read to ensure immersion in the data and to identify initial patterns related to children's environmental engagement. Second, meaningful segments of data were coded to capture children's emotional, cognitive, and behavioral expressions. Third, codes were systematically organized into broader categories reflecting conceptual similarities across affective and behavioral dimensions. Fourth, preliminary themes were reviewed and refined through continuous comparison with the original data to ensure coherence and alignment with the study objectives. Fifth, themes were defined and named in accordance with the study's conceptual framework, resulting in eight main categories under the CEADS and CEBS domains. Finally, in the sixth stage, a comprehensive narrative report was produced, supported by representative excerpts from participants and, where appropriate, simple frequencies and percentages to enhance interpretation of qualitative patterns. To ensure the trustworthiness of the analysis, coding and theme development were independently reviewed by two researchers, yielding an inter-rater agreement of 0.96, indicating a high level of reliability. Furthermore, triangulation between quantitative and qualitative findings strengthened the overall validity of the study by providing a more comprehensive interpretation of the intervention's effects.

### 3.10 Ethics approval and consent to participate

Ethical approval for this study was granted by the Research Ethics Committee at King Faisal University (Approval No. KFU-REC-2025-November-EA000953, dated November 10, 2025), in accordance with national and international ethical standards for research involving children. Written informed consent for the participation of the 250 children was obtained from their parents, who were provided with a clear and detailed explanation of the study's objectives, procedures, potential benefits, and the voluntary nature of participation. Parents were also explicitly informed of their right to withdraw their children from the study at any stage without any consequences. To ensure confidentiality and protect participants' privacy, all identifying information was removed from the dataset, and anonymized codes were used throughout the research process. In addition, all data were securely stored and accessible only to the research team. The study was conducted in full compliance with ethical principles to safeguard the safety, dignity, and wellbeing of all participants throughout the research process.

## 4 Results

### 4.1 Results of the first research question

The first research question examined whether project-based environmental learning within the STEM framework leads to statistically significant improvements in children's environmental knowledge related to the Sustainable Development Goals (SDGs) compared with conventional teaching methods. To address this question, scores obtained on the Environmental Knowledge Test were analyzed using both descriptive and inferential statistical procedures. Descriptive statistics were calculated to summarize participants' performance, whereas independent-samples *t*-tests were conducted to examine differences between the experimental and control groups. Prior to analysis, the assumptions of normality and homogeneity of variance were examined and found to be satisfactory. The results are presented in Table 3.

**Table 3:** Independent samples t-test results for students' environmental knowledge.

Cognitive domain	Group	*N*	Mean	SD	*t*	df	*p*	95% CI (mean difference)	*η^2^*
Remembering	Experimental	125	8.92	0.88	6.84	248	< 0.001	[1.58, 2.84]	0.16
	Control	125	6.71	1.02					
Understanding	Experimental	125	7.45	0.91	8.27	248	< 0.001	[1.89, 3.00]	0.22
	Control	125	5.02	1.14					
Application	Experimental	125	6.88	0.79	9.63	248	< 0.001	[2.19, 3.35]	0.27
	Control	125	4.11	1.08					
Overall Knowledge	Experimental	125	23.25	1.85	10.54	248	< 0.001	[6.20, 8.82]	0.31
	Control	125	15.84	2.21					

The total score of the environmental knowledge test is 25 marks, distributed across three domains: remembering (10 marks), understanding (8 marks), and application (7 marks). Mean scores are interpreted relative to the maximum possible score for each domain.

The level of statistical significance was set at *p* = 0.05.

The results presented in Table 3 indicate a consistent and statistically significant advantage for the experimental group across all cognitive domains of environmental knowledge (*p* < 0.001). In addition, the observed effect sizes were large according to conventional benchmarks, suggesting that the differences between the experimental and control groups were not only statistically significant but also educationally meaningful. Taken together, these findings provide strong evidence that project-based environmental learning within the STEM framework exerted a substantial positive influence on children's environmental knowledge.

A closer examination of the individual cognitive domains reveals a progressive pattern of improvement. In the remembering domain, children in the experimental group achieved significantly higher scores than their counterparts in the control group, with a large effect size (η^2^ = 0.16). This finding suggests that participation in environmental projects enhanced children's ability to retain and recall essential environmental concepts, thereby strengthening foundational cognitive processes necessary for subsequent learning.

Building upon these gains in basic knowledge acquisition, even stronger effects emerged in higher cognitive domains. Specifically, the understanding domain yielded a larger effect size (η^2^ = 0.22), indicating that the intervention facilitated deeper conceptual comprehension and enabled children to interpret environmental issues within meaningful real-life contexts. Furthermore, the strongest effect among the individual domains was observed in the application domain (η^2^ = 0.27), suggesting that the intervention was particularly effective in supporting children's ability to transfer environmental knowledge to practical situations and problem-solving tasks. This pattern of findings is consistent with the emphasis placed by STEM-based learning approaches on authentic learning experiences, experiential engagement, and the application of knowledge in real-world contexts.

The cumulative impact of these improvements is reflected in the overall environmental knowledge score, where the experimental group significantly outperformed the control group, with a large effect size (η^2^ = 0.31). This result indicates that the intervention generated broad-based cognitive gains extending across all measured dimensions of environmental knowledge rather than producing isolated improvements in specific domains. Such a pattern strengthens the conclusion that the intervention contributed to comprehensive cognitive development related to environmental learning.

Although the independent-samples *t*-test results provide compelling evidence of the intervention's effectiveness, the hierarchical structure of the dataset required additional examination. Because the participating children were nested within 12 educational institutions, it was necessary to determine whether the observed intervention effect remained stable after accounting for potential clustering at the institutional level. Consequently, a multilevel analytical approach was employed to evaluate the robustness of the findings while controlling for contextual variation across institutions.

Accordingly, a two-level linear mixed-effects model was estimated, with children specified at Level 1 and educational institutions at Level 2. Instructional group (experimental vs. control) was entered as a fixed effect, whereas institutional differences were modeled through random intercepts. The results of this analysis are presented in Table 4.

**Table 4:** Two-level linear mixed-effects model predicting environmental knowledge.

Effect	Estimate	SE	*t*	*p*
Intervention (experimental vs. control)	7.21	0.68	10.60	< 0.001
Random intercept variance (institution level)	0.42			
Residual variance (student level)	4.83			
Intraclass correlation coefficient (ICC)	0.08			

Table 4 shows that the instructional intervention remained a statistically significant predictor of children's environmental knowledge after controlling for institutional clustering effects (*p* < 0.001). The estimated intervention effect (β = 7.21) indicates that children who participated in the STEM-based project intervention achieved substantially higher environmental knowledge scores than those who received conventional instruction. This finding converges with the results of the independent-samples *t*-tests and provides additional evidence supporting the robustness of the intervention effect across educational settings.

The intraclass correlation coefficient (ICC = 0.08) indicates that approximately 8% of the variance in environmental knowledge scores was attributable to differences between institutions, whereas 92% was attributable to variation among individual children. Although the institutional-level variance was relatively modest, the ICC confirms the presence of a hierarchical data structure and therefore justifies the use of multilevel modeling.

Taken together, the findings demonstrate that project-based environmental learning within the STEM framework produced a consistent and robust positive effect on children's environmental knowledge. Importantly, the intervention effect remained statistically significant even after accounting for institutional differences, indicating that the observed gains were not confined to specific educational settings. Moreover, the pattern of results suggests that the intervention was particularly effective in promoting higher-order cognitive development, as evidenced by the strongest gains observed in the application domain. These findings provide strong evidence that project-based environmental learning within STEM can facilitate meaningful knowledge acquisition and support the transfer of environmental learning to authentic contexts related to sustainability and the SDGs.

### 4.2 Results of the second research question?

The second research question examined whether there were statistically significant differences in children's environmental affective domain related to sustainability attributable to the teaching method (project-based environmental learning within the STEM framework vs. conventional instruction). To address this question, scores obtained on the Environmental Affective Domain Scale were analyzed using both descriptive and inferential statistical procedures. Descriptive statistics were computed to summarize participants' performance, whereas independent-samples *t*-tests were conducted to examine differences between the experimental and control groups. Prior to analysis, the assumptions of normality and homogeneity of variance were examined and found to be satisfactory. The results are presented in Table 5.

**Table 5:** Independent-samples *t*-test results for children's environmental affective domain.

Affective domain	Group	*N*	Mean	SD	*t*	df	*p*	95% CI (mean difference)	*η^2^*
Environmental care and cleanliness	Experimental	125	20.96	1.85	15.38	248	< 0.001	[3.37, 4.35]	0.49
	Control	125	17.10	2.10					
Individual responsibility	Experimental	125	21.48	1.78	17.91	248	< 0.001	[4.03, 5.03]	0.56
	Control	125	16.95	2.20					
Participation and collaboration	Experimental	125	18.74	1.66	16.19	248	< 0.001	[3.36, 4.28]	0.51
	Control	125	14.92	2.05					
Pride and satisfaction	Experimental	125	19.15	1.52	21.18	248	< 0.001	[4.57, 5.51]	0.64
	Control	125	14.11	2.19					
Overall affective score	Experimental	125	80.33	5.40	23.26	248	< 0.001	[15.79, 18.71]	0.69
	Control	125	63.08	6.30					

The environmental affective domain scale consists of a total score of 90 points, distributed across four domains: environmental care and cleanliness (24), individual responsibility (24), participation and collaboration (21), and pride and satisfaction in environmental actions (21).

The results presented in Table 5 indicate a consistent and statistically significant advantage for the experimental group across all dimensions of the environmental affective domain (*p* < 0.001). Furthermore, all effect sizes were large according to conventional benchmarks (η^2^ = 0.49–0.69), indicating that the observed differences were not only statistically significant but also educationally meaningful. Collectively, these findings suggest that project-based environmental learning within the STEM framework had a substantial positive impact on children's environmental affective development.

A closer examination of the individual dimensions reveals a consistent pattern of improvement across the affective domain. Children in the experimental group demonstrated higher levels of environmental care and cleanliness, stronger feelings of personal responsibility, greater willingness to participate and collaborate in environmental activities, and higher levels of pride and satisfaction associated with environmentally responsible actions. These findings suggest that the intervention extended beyond the acquisition of environmental awareness and contributed to the development of environmentally responsible attitudes and values.

Taken together, the pattern of results suggests a progressive strengthening of affective engagement, beginning with environmental care and responsibility and extending toward collaboration, emotional attachment, and satisfaction derived from environmental actions. Rather than influencing a single isolated aspect of children's environmental attitudes, the intervention appears to have fostered multiple interconnected dimensions of affective development. This pattern reflects the integrated nature of project-based environmental learning, in which emotional engagement, social interaction, and personal responsibility are simultaneously cultivated through authentic learning experiences.

Although the independent-samples *t*-test results provide strong evidence of the intervention's effectiveness, the hierarchical nature of the dataset warranted further examination. Because the participating children were nested within 12 educational institutions, a multilevel analytical approach was employed to determine whether the observed effects remained stable after accounting for potential institutional clustering. Accordingly, a two-level linear mixed-effects model was estimated, with children specified at Level 1 and institutions at Level 2. Instructional group (experimental vs. control) was entered as a fixed effect, whereas institutional variability was modeled using random intercepts. The results are presented in Table 6.

**Table 6:** Two-level linear mixed-effects model predicting children's environmental affective outcomes.

Effect	Estimate	SE	*t*	*p*
Intervention (experimental vs. control)	16.84	1.52	11.08	< 0.001
Random intercept variance (institution level)	0.48			
Residual variance (child level)	4.85			
Intraclass correlation coefficient (ICC)	0.09			

Table 6 shows that the instructional intervention remained a statistically significant predictor of children's environmental affective outcomes after controlling for institutional clustering effects (*p* < 0.001). The estimated intervention effect (β = 16.84) indicates that children who participated in the STEM-based project intervention achieved substantially higher affective domain scores than those who received conventional instruction. This finding converges with the results of the independent-samples *t*-tests and provides additional evidence supporting the robustness of the intervention effect across educational settings.

The intraclass correlation coefficient (ICC = 0.09) indicates that approximately 9% of the variance in environmental affective outcomes was attributable to differences between institutions, whereas 91% was attributable to variation among individual children. Although the institutional-level variance was relatively modest, the ICC confirms the presence of a hierarchical data structure and therefore justifies the use of multilevel modeling.

Overall, the convergence of findings from both the independent-samples *t*-tests and the multilevel model provides strong and consistent evidence that project-based environmental learning within the STEM framework meaningfully enhances children's environmental affective development. The relatively small institutional variance suggests that the observed effects were primarily associated with the instructional approach rather than with differences across participating institutions. Consequently, the findings strengthen the internal validity of the study and support the conclusion that the intervention was effective in fostering positive environmental attitudes, responsibility, collaboration, and emotional engagement with sustainability-related practices.

To complement these quantitative findings, the following section presents qualitative results from semi-structured interviews, providing deeper insight into children's emotional engagement and lived experiences within the environmental affective domain.

#### 4.2.1 Environmental care and cleanliness

The results indicate a statistically significant advantage for the experimental group over the control group in environmental care and cleanliness, with a large effect size (η^2^ = 0.49). This finding reflects a clear improvement in children's emotional sensitivity and affective orientation toward maintaining environmental cleanliness as a result of the intervention. To deepen the interpretation of these findings, qualitative data from the interviews provided complementary evidence of strong emotional engagement with cleanliness practices. A high proportion of children in the experimental group (118 out of 125; 94.4%) demonstrated heightened sensitivity and active behavioral responses toward maintaining cleanliness. Their responses reflected not only awareness but also emotional concern and proactive engagement:

“*I feel very sad when I see garbage in the school yard because it makes the place look ugly, and I always want to clean it even if I am not the one who caused it.”* (Participant 3 [P3])

“*When I see someone throwing trash on the ground, I feel it is wrong, and I try to kindly tell them that our school should stay clean.”* (P5)

“*I cannot ignore trash, so I collect it myself because I feel the place becomes better when it is clean.”* (P120)

“*I like when our classroom and school yard are always clean because cleanliness makes us feel comfortable and respectful toward the place.”* (P87)

“*When we participate in cleaning activities at school, I feel I am helping make the environment better for everyone.”* (P13)

These excerpters indicate a clear transition from passive awareness to emotionally driven action. Cleanliness is no longer perceived as a rule to follow but as a shared value associated with belonging, respect, and collective responsibility. This emotional internalization explains the higher performance of the experimental group and confirms the effectiveness of project-based STEM learning in strengthening environmental sensitivity.

#### 4.2.2 Individual responsibility

The findings showed a statistically significant difference in favor of the experimental group, with a large effect size (η^2^ = 0.56). The qualitative data further support this interpretation by revealing a shift from externally guided behavior to internally regulated responsibility. A large proportion of children in the experimental group (115 out of 125; 92%) demonstrated a strong sense of personal accountability in their daily environmental actions. Their responses illustrate this internalization:

“*I always turn off the lights when I leave the room because electricity is important and should not be wasted.”* (P2)

“*I remind my family that water is a blessing and we should protect it because it is essential for life.”* (P23)

“*I like to protect the environment. It is not only adults who are responsible, but all of us who live on this Earth.”* (P45)

“*When I forget to turn off the light, I feel I did something wrong and try not to repeat it again.”*

“*Sometimes I reduce my use of things because I think about the future and the environment.”* (P124)

These responses indicate the emergence of self-regulated environmental behavior guided by internal moral reasoning rather than external instruction. Children demonstrate growing environmental conscience, reflecting responsibility as a stable personal value rather than situational compliance.

#### 4.2.3 Participation and collaboration

The results indicate a statistically significant advantage for the experimental group compared with the control group in participation and collaboration, with a strong effect size (η^2^ = 0.51). The qualitative findings further reinforce this result, showing that a high proportion of children in the experimental group (117 out of 125; 93.6%) actively engaged in collaborative environmental practices not only within school settings but also in family contexts. Their responses highlight the social and interactive dimension of learning.

“*I like working with my friends to plant trees and clean the school because we feel we are achieving something important together.”* (P1)

“*When we work as a team, I feel happy because everyone participates and the work becomes faster and more enjoyable.”* (P9)

“*I always encourage my classmates to join environmental activities because teamwork has a greater impact.”* (P94)

“*I talk with my family about environmental activities, and sometimes we apply the same ideas at home.”* (P101)

“*I feel cooperation makes us stronger in protecting the environment because we help each other.”* (P113)

These excerpts demonstrate that environmental participation evolved into a socially reinforced practice characterized by motivation, shared responsibility, and transfer of learning beyond the classroom. The findings confirm that STEM-based project learning effectively strengthens collaborative engagement and extends environmental action into everyday life contexts.

#### 4.2.4 Pride and satisfaction

A statistically significant difference was found in favor of the experimental group compared to the control group, with a large effect size (η^2^ = 0.64). Qualitative interview data supported this finding, indicating high levels of emotional fulfillment among children in the experimental group. Most participants (93.6%) reported feelings of pride and satisfaction related to their environmental actions, reflecting strong emotional engagement with the activities. The following quotations illustrate these positive emotional responses:

“*I feel very proud when I plant a tree and see it growing day by day because I feel I am leaving a positive mark in nature.”* (P7)

“*When my teacher or friends praise me after an environmental activity, I feel my work is truly valuable.”* (P33)

“*I feel very happy when I see that my school becomes cleaner because of my participation.”*

“*Every time I finish an environmental activity, I feel I have done something meaningful and want to repeat it.”* (P69)

“*I like participating in environmental activities because I feel I am helping the Earth and making it better.”* (P114)

These quotations indicate that pride and satisfaction were not merely momentary emotions, but sustained affective responses shaped by visible outcomes, social recognition, and active participation. The combination of hands-on experience and observable environmental improvement contributed to the internalization of positive emotional reinforcement. Consequently, environmental engagement became a source of self-worth and intrinsic motivation rather than a routine school requirement. This convergence between quantitative and qualitative evidence reinforces the robustness of the findings and confirms the effectiveness of the STEM-based project approach in fostering enduring positive environmental affect.4.3 Results of the third research question

The third research question examined whether there were statistically significant differences in children's environmental behavior scores related to sustainability attributable to the teaching method (project-based environmental learning within the STEM framework compared with conventional instruction). To address this question, data from the Environmental Behavioral Domain Scale were analyzed using descriptive and inferential statistical procedures. The results are presented in Table 7, which provides a comprehensive comparison between the two groups across all behavioral domains.

**Table 7:** Independent samples *t*-test results for children's environmental behavioral domain.

Environmental behavioral domain	Group	*N*	Mean	SD	*t*	df	*p*	95% CI	*η^2^*
Water, energy, and waste management	Experimental	125	21.70	1.85	6.95	248	< 0.001	[2.60, 4.20]	0.16
	Control	125	17.80	2.10					
Participation in environmental activities	Experimental	125	22.10	1.65	8.40	248	< 0.001	[3.80, 5.60]	0.22
	Control	125	16.90	2.25					
Awareness of environmental activity importance	Experimental	125	19.00	1.75	7.60	248	< 0.001	[2.70, 4.50]	0.19
	Control	125	15.40	2.05					
Initiative for environmental actions	Experimental	125	18.60	1.70	8.90	248	< 0.001	[3.20, 5.20]	0.24
	Control	125	14.30	2.20					
Overall behavioral score	Experimental	125	81.40	5.60	9.85	248	< 0.001	[12.20, 20.80]	0.30
	Control	125	64.90	6.40					

The environmental behavioral domain scale consists of a total score of 90 points distributed across four sub-domains: water, energy, and waste management (24), participation in environmental activities (24), awareness of environmental activity importance (21), and initiative for environmental actions (21).

The results presented in Table 7 indicate a consistent and statistically significant difference between the experimental and control groups across all environmental behavioral domains (all *p* < 0.001). All effect sizes were large according to conventional benchmarks (η^2^ ≥ 0.14), indicating that the observed differences were not only statistically significant but also educationally meaningful. Importantly, all behavioral dimensions consistently favored the experimental group, suggesting that project-based environmental learning within the STEM framework was associated with improved environmental behavioral outcomes among children.

Across the specific domains, relatively stronger effects were observed in Participation in Environmental Activities and Initiative for Environmental Actions, indicating enhanced engagement in active and self-directed environmental behaviors. Substantial effects were also found in Water, Energy, and Waste Management as well as Awareness of Environmental Activity Importance, suggesting that the intervention supported both practical environmental actions and conceptual awareness of sustainability-related behaviors.

Overall, the pattern of findings suggests that the intervention was associated with meaningful improvements across all behavioral dimensions rather than isolated behavioral changes. The consistency of large effect sizes across subdomains further supports the practical relevance of the intervention in promoting environmentally responsible behavior in early childhood settings.

Although the independent samples *t*-test results provide strong evidence of group differences, the hierarchical structure of the data required further analysis. Since children were nested within 12 educational institutions, a multilevel analytical approach was applied to account for potential clustering effects and to examine the robustness of the observed relationships across different educational contexts.

Accordingly, a two-level linear mixed-effects model was estimated, with students at Level 1 and institutions at Level 2. Instructional group was included as a fixed effect, while institutional differences were modeled using random intercepts. The results are presented in Table 8.

**Table 8:** Two-level linear mixed-effects model predicting environmental affective outcomes.

Effect	Estimate	SE	*t*	*p*
Intervention (experimental vs. control)	6.85	0.72	9.51	< 0.001
Random intercept variance (institution level)	0.48	—	—	—
Residual variance (child level)	5.21	—	—	—
Intraclass correlation coefficient (ICC)	0.09	—	—	—

Table 8 shows that the instructional intervention remained a statistically significant predictor of children's environmental behavior after accounting for institutional clustering (*p* < 0.001). The estimated effect (β = 6.85) indicates that children in the experimental group achieved higher environmental behavior scores compared with those in the control group.

The intraclass correlation coefficient (ICC = 0.09) indicates that approximately 9% of the variance in environmental behavior was attributable to differences between institutions, while 91% was attributable to individual differences among children. This confirms a modest clustering effect and justifies the use of multilevel modeling.

Importantly, the persistence of the intervention effect after controlling for institutional variation strengthens the robustness of the findings. The convergence between the independent samples *t*-tests and the multilevel model provides consistent evidence that project-based environmental learning within the STEM framework is associated with improved environmental behavior among children. However, these findings should be interpreted as context-bound educational effects rather than strictly causal long-term behavioral transformations.

To further enrich these quantitative findings, the following section presents qualitative results derived from semi-structured interviews, organized according to the main environmental behavioral domains.

#### 4.3.1 Water, energy, and waste management

A statistically significant difference was found in favor of the experimental group compared with the control group, with a large effect size (η^2^ = 0.16), indicating improved resource management behaviors following the intervention. Qualitative data further support this result, showing that a high proportion of children in the experimental group (118 out of 125; 94.4%) demonstrated consistent sustainable practices in their daily routines. Their responses reflect the internalization of environmental behaviors.

“*I always try to save water when washing my hands.”* (P7)

“*I turn off the lights when leaving the room.”* (P9)

“*I think before throwing things away and try to recycle.”* (P13)

“*I remind my family not to waste water.”* (P29)

“*I reuse paper instead of wasting it.”* (P33)

“*I separate waste at home whenever possible.”* (P97)

These responses indicate a shift from environmental awareness to habitual behavioral practice, suggesting that STEM-based project learning supported the internalization of sustainability principles in everyday life.

#### 4.3.2 Participation in environmental activities

A statistically significant difference was observed in favor of the experimental group compared with the control group, with large effect size (η^2^ = 0.22), indicating higher levels of participation in environmental activities following the intervention. Qualitative findings further supported these results, revealing that a high proportion of children in the experimental group (113 out of 125; 90.4%) actively engaged in environmental activities in both school and home contexts, reflecting sustained participation beyond the immediate learning environment. Children's responses included the following:

“*I help clean the classroom with my classmates.”* (P13)

“*I join tree-planting activities at school.”* (P52)

“*I take part in recycling activities.”* (P55)

“*I collect litter in the school yard.”* (P81)

“*I participate in environmental campaigns.”* (P92)

“*I encourage my classmates to join environmental work.”* (P103)

“*I recycle at home with my family.”* (P111)

These findings indicate that environmental participation extended beyond structured activities to voluntary and family-based engagement, reflecting strengthened collective responsibility.

#### 4.3.3 Awareness of environmental activity importance

A statistically significant difference was found in favor of the experimental group compared with the control group, with a large effect size (η^2^ = 0.19), indicating higher levels of environmental awareness following the intervention. Qualitative findings further supported these results, revealing that 115 children (92%) developed a clear understanding of the importance of environmental protection and sustainability. Children's responses reflected the following:

“*Environmental activities protect nature and reduce pollution.”* (P23)

“*We must protect the environment for future generations.”* (P59)

“*Small actions like recycling make a big difference.”* (P62)

“*Everyone is responsible for the environment.”* (P73)

“*My actions affect the environment.”* (P84)

“*Recycling protects natural resources.”* (P109)

“*Protecting the environment is necessary for life.”* (P121)

These responses indicate the development of structured environmental understanding and awareness of human-environment interdependence.

#### 4.3.4 Initiative for environmental actions

Finally, a statistically significant difference was found in favor of the experimental group compared with the control group, with a large effect size (η^2^ = 0.24), indicating stronger initiative in environmental actions following the intervention. Qualitative findings further supported these results, revealing that 111 children (88.8%) demonstrated proactive environmental behaviors without external prompting, reflecting emerging environmental agency. Children's responses reflected the following:

“*I clean the classroom or yard without being asked.”* (P5)

“*I pick up litter when I see it.”* (P41)

“*I remind others to care about the environment.”* (P51)

“*I organize small cleaning activities at home.”* (P69)

“*I act immediately when I see environmental problems.”* (P99)

“*I recycle without being told.”* (P118)

“*I encourage others to protect the environment.”* (P119)

These findings indicate a shift from guided behavior to self-initiated environmental action, reflecting the development of autonomy and environmental responsibility. Overall, the integration of findings across all behavioral domains demonstrates that project-based environmental learning within the STEM framework not only enhances environmental knowledge and awareness but also leads to sustained and meaningful behavioral transformation. This transformation reflects a progressive shift from understanding to practice, and ultimately to self-directed environmental responsibility.

## 5 Discussion

The results of the first research question indicated that project-based environmental learning within the STEM framework significantly improved children's environmental knowledge compared to traditional instruction across the levels of remembering, understanding, and application. This finding suggests that the intervention had a comprehensive rather than a fragmented impact on cognitive development, leading to more integrated and meaningful knowledge construction. This outcome can be attributed to the nature of project-based STEM learning, which emphasizes active engagement and direct experimentation within authentic environmental contexts, thereby positioning children as active constructors of knowledge rather than passive recipients.

This interpretation aligns with Constructivist Theory, which posits that knowledge is actively constructed through interaction with the environment rather than transmitted directly (Bybee, 2013; Bani Irshid et al., 2023; Khasawneh et al., 2022; Taber, 2024). It is further supported by Kolb's Experiential Learning Theory, which emphasizes the cyclical process of concrete experience, reflection, conceptualization, and active experimentation as a mechanism for deep learning (Al-Barakat et al., 2026; Khasawneh et al., 2023). Similarly, previous studies (Abuodeh and Abumousa, 2021; Awamleh, 2024; Hawamdeh et al., 2025) underscore the importance of exploratory and hands-on learning in fostering cognitive growth during early childhood. Nevertheless, the observed improvement may be partially influenced by the novelty of the intervention, which warrants caution in generalizing the findings beyond the study context.

Building on this cognitive development, the results of the second research question revealed a marked improvement in children's environmental affective outcomes, including environmental care, personal responsibility, cooperation, and satisfaction with environmental practices. This suggests that the shift from knowledge to attitudes was not automatic but was facilitated through meaningful, socially interactive learning experiences embedded within the STEM-based project framework.

The findings further demonstrated consistently large effect sizes across all affective dimensions, indicating a strong educational impact of the intervention. This can be attributed to the nature of project-based STEM learning, which provides rich, authentic, and experiential learning environments in which children actively engage in real-world environmental tasks. Such engagement fosters emotional connections to environmental issues and transforms abstract knowledge into stable attitudes and value-based dispositions.

In addition, collaborative group work, role distribution, and shared decision-making processes appear to have strengthened children's sense of responsibility, ownership, and belonging. These social and participatory elements likely contributed to the substantial magnitude of the observed effects by encouraging learners to internalize environmental values through practice rather than passive reception.

From a pedagogical perspective, the strong effect sizes suggest that the intervention extended beyond cognitive acquisition to the reshaping of children's affective dispositions. By integrating experiential learning, social interaction, and hands-on application, the STEM-based project approach created conditions particularly conducive to affective change. This may explain why the affective outcomes demonstrated stronger-than-expected effects, as attitudes and values are typically more responsive to immersive and meaningful learning experiences than to traditional instructional methods.

Moreover, this finding is supported by Affective Learning Theory, which emphasizes that values and attitudes are more effectively developed through socially meaningful and experiential learning environments. This theoretical perspective further reinforces the interpretation that the observed improvements in children's affective outcomes are a direct result of engaging, collaborative, and experience-based learning processes embedded within the STEM-based project approach (Al-Hassan et al., 2012; Bani Irshid et al., 2023; Bataineh et al., 2025).

Self-Determination Theory further explains these improvements by highlighting the role of autonomy, competence, and relatedness in enhancing intrinsic motivation and sustaining positive affective engagement (AlAli and Al-Barakat, 2024; Ryan and Deci, 2023; Yang et al., 2025). In addition, Place Attachment Theory suggests that sustained and direct interaction with the environment strengthens emotional bonds and increases environmental sensitivity (Al-Barakat et al., 2026; Awamleh, 2024; Al-Abdullatif and Gameil, 2021; Alta'ban and Naji, 2020; Lengyel, 2020; Chang et al., 2026; Xie and Wang, 2024).

However, these findings should be interpreted with caution, as contextual factors such as teacher practices, classroom dynamics, and social desirability bias may have influenced students' responses and potentially limited the stability of attitudinal change over time.

Extending this trajectory from cognition and affect to behavior, the results of the third research question demonstrated a significant improvement in children's environmental behavior, including water, energy, and waste management, participation in environmental activities, and environmental initiative. This behavioral development reflects a progressive transition from knowledge and attitudes toward practice; however, it should not be interpreted as a direct or linear causal effect, but rather as the outcome of dynamic interactions among learning experiences, classroom environment, and social context.

These findings are consistent with the Theory of Planned Behavior, which posits that behavior is shaped by attitudes, subjective norms, and perceived behavioral control (Ho et al., 2024). They are also supported by Social Learning Theory, which emphasizes modeling, observation, and imitation as key mechanisms for behavioral acquisition in social settings (Bataineh et al., 2025; Fraihat et al., 2022; Morris, 2020; Villarroel Henríquez et al., 2025). Furthermore, Kolb's Experiential Learning Theory explains this progression through iterative cycles of experience and application. Environmental psychology also supports this interpretation by highlighting that direct awareness of behavioral consequences fosters responsible environmental actions (Suherman et al., 2025). However, the behavioral outcomes should be critically interpreted in light of two key limitations: the possibility of guided compliance with teacher expectations rather than internalized behavioral change, and the limited transferability of behaviors beyond the structured classroom context (Al-Barakat et al., 2023; Abanoz and Yabaş, 2025; Bataineh et al., 2026; Suherman et al., 2025).

From a critical integrative perspective, the overall findings indicate that project-based environmental learning within the STEM framework fosters a gradual and interconnected developmental trajectory beginning with knowledge, extending to attitudes, and culminating in behavior. However, this sequence should not be interpreted as linear or deterministic, but rather as the product of a complex interaction among instructional design, experiential learning processes, developmental characteristics of early childhood learners, and classroom context. At this stage of development, learning outcomes are strongly influenced not only by instructional content but also by interaction quality, teacher mediation, and the social environment, making the cognitive–affective–behavioral transition a multifactorial rather than a mechanistic process.

Moreover, the results are likely influenced by methodological and contextual factors that should be considered when interpreting the findings. Variations in teacher performance and implementation fidelity may have contributed to differences in learning experiences across classrooms, suggesting that the observed effects may not be fully uniform. In addition, the novelty effect and the Hawthorne effect may partially explain the observed improvements, as children's awareness of participating in a new and observed educational intervention may temporarily enhance engagement and positive responses. Accordingly, the findings should be interpreted as context-bound and short-term educational effects rather than stable or long-term transformations, highlighting the need for longitudinal research to examine their sustainability.

Despite these limitations, the study offers several significant contributions. Contextually, it provides empirical evidence from early childhood education in Saudi Arabia, a setting that remains underrepresented in STEM and environmental education research. Methodologically, it integrates cognitive, affective, and behavioral dimensions within a single unified intervention rather than treating them as isolated outcomes. Conceptually, it advances sustainability education by operationalizing it as a measurable learning outcome aligned with the Sustainable Development Goals (SDGs), through the integration of STEM education, project-based learning, and environmental education within a coherent pedagogical framework.

Overall, the findings suggest that project-based environmental learning within the STEM framework is an effective approach for enhancing environmental learning outcomes across cognitive, affective, and behavioral domains in early childhood. However, these findings should be interpreted as context-dependent educational effects rather than stable developmental transformations, with further longitudinal research required to establish their long-term validity and sustainability.

## 6 Conclusions, recommendations, limitations, and future research directions

Building on the findings of the present study, it can be concluded that project-based environmental learning within the STEM framework represents an effective pedagogical approach for enhancing children's environmental learning outcomes in a comprehensive and integrated manner. The results indicate statistically significant short-term improvements in environmental knowledge, affect, and behavior following the intervention, suggesting that learning is more effectively developed when embedded in authentic, real-life, and experiential contexts rather than through abstract or traditional instructional methods.

Extending this conclusion, the findings further suggest that the pedagogical value of this approach lies in its capacity to actively engage children in meaningful environmental experiences that connect learning with action. Through structured participation in inquiry-based and collaborative tasks, children are supported in developing initial forms of environmental responsibility and engagement. This indicates a clear shift in learning orientation from passive knowledge acquisition toward active, situated learning that is grounded in real-world environmental contexts relevant to early childhood experiences.

In light of these outcomes, the study recommends the systematic integration of project-based environmental learning within STEM curricula at the early childhood level. Such integration should prioritize authentic learning experiences that connect classroom content to real environmental issues. In addition, instructional practices should emphasize inquiry, collaboration, and hands-on engagement to strengthen learning effectiveness. To support successful implementation, targeted professional development programs are essential to equip teachers with the pedagogical skills required for integrating STEM and environmental education effectively. Furthermore, extending learning activities beyond the classroom through school–home and community partnerships is strongly recommended to reinforce learning continuity and contextual application.

Despite the promising results, several limitations must be acknowledged in order to ensure a balanced interpretation of the findings. First, the study was conducted within a single geographical region, which may limit the external validity and broader generalizability of the results. Second, the reliance on structured instruments and interview data without incorporating systematic naturalistic observations may restrict the depth of behavioral interpretation in authentic settings. Third, the study focused exclusively on children as participants, without including teachers or caregivers, whose roles are critical in shaping and sustaining environmental learning processes. These limitations suggest that the findings should be interpreted within the specific contextual and methodological boundaries of the study.

Building upon these limitations, future research is encouraged to expand the scope of investigation to include diverse educational systems and cultural contexts, particularly in public and underrepresented school settings. Incorporating multiple stakeholders such as teachers and parents would provide a more holistic understanding of how environmental learning is supported and sustained across different environments. In addition, longitudinal research designs are strongly recommended to examine whether the observed short-term improvements persist, decline, or evolve into stable behavioral patterns over time. Moreover, the use of mixed-method approaches that integrate observational data with quantitative and qualitative measures would strengthen methodological rigor and provide deeper insight into actual behavioral enactment. Comparative studies across different pedagogical models and developmental stages would also contribute to a more nuanced understanding of the effectiveness and adaptability of STEM-based environmental learning.

## Data Availability

The raw data supporting the conclusions of this article will be made available by the authors, without undue reservation.
